# Supplementary material for: Functional partitioning of lipoic acid decouples cellular abundance from mitochondrial utilization
Source: bioRxiv. 2026 May 23:2026.05.22.727209. Preprint. [Version 1] doi: 10.64898/2026.05.22.727209 (PMC13228248; doi:10.64898/2026.05.22.727209)

bioRxiv preprint doi: https://doi.org/10.64898/2026.05.22.727209; this version posted May 23, 2026. The copyright holder for this preprint (which was not certified by peer review) is the author/funder, who has granted bioRxiv a license to display the preprint in perpetuity. It is made available under a CC-BY 4.0 International license.

Supplemental Figure 1.

(A-H) Representative extracted ion chromatograms of derivatized LA-d_5 internal standard from the denoted cell lines. (I) Quantification of the mean response ratio of derivatized LA/LA-d_5 sum area $\pm$ SEM from the denoted cell lines.

Supplemental Figure 2.

(A) Standard curve of the response ratio of derivatized LA/LA-d_5 detected by Orbitrap LC-MS. (B-H) Relative counts per well of control (B, E, G), MCAT-deficient (C,F), and MECR-deficient (D,H) C2C12, H9c2, or HeLa cells cultured in an IncuCyte® system supplemented with vehicle or LA for 3-4 days. Data are mean relative cell counts per well; error bars are SEM from n = 4 technical replicates.

Supplemental Figure 1

bioRxiv preprint doi: https://doi.org/10.64898/2026.05.22.727209; this version posted May 23, 2026. The copyright holder for this preprint (which was not certified by peer review) is the author/funder, who has granted bioRxiv a license to display the preprint in perpetuity. It is made available under a CC-BY 4.0 International license.

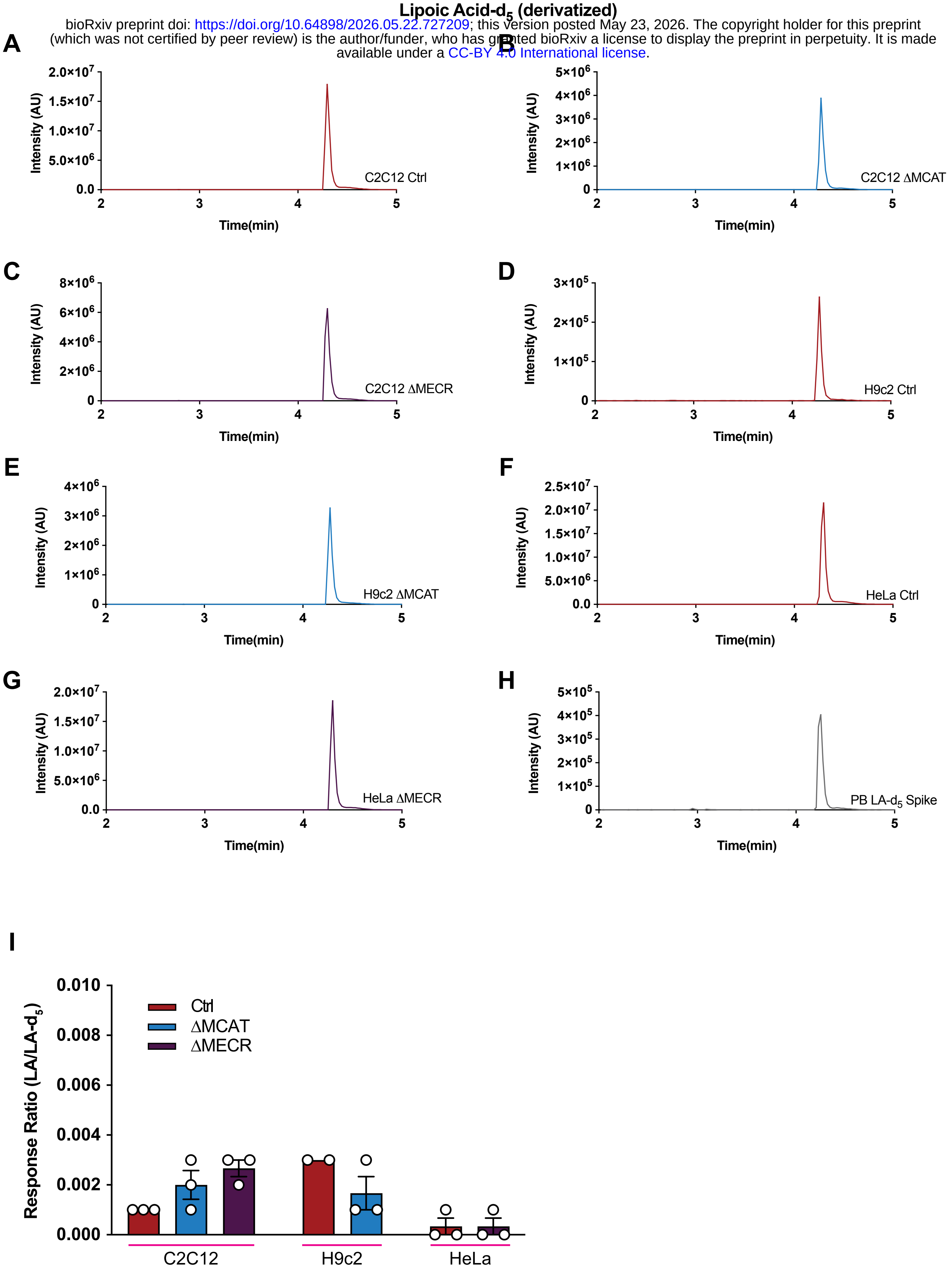

Supplemental Figure 2

bioRxiv preprint doi: https://doi.org/10.64898/2026.05.22.727209; this version posted May 23, 2026. The copyright holder for this preprint (which was not certified by peer review) is the author/funder, who has granted bioRxiv a license to display the preprint in perpetuity. It is made available under a CC-BY 4.0 International license.

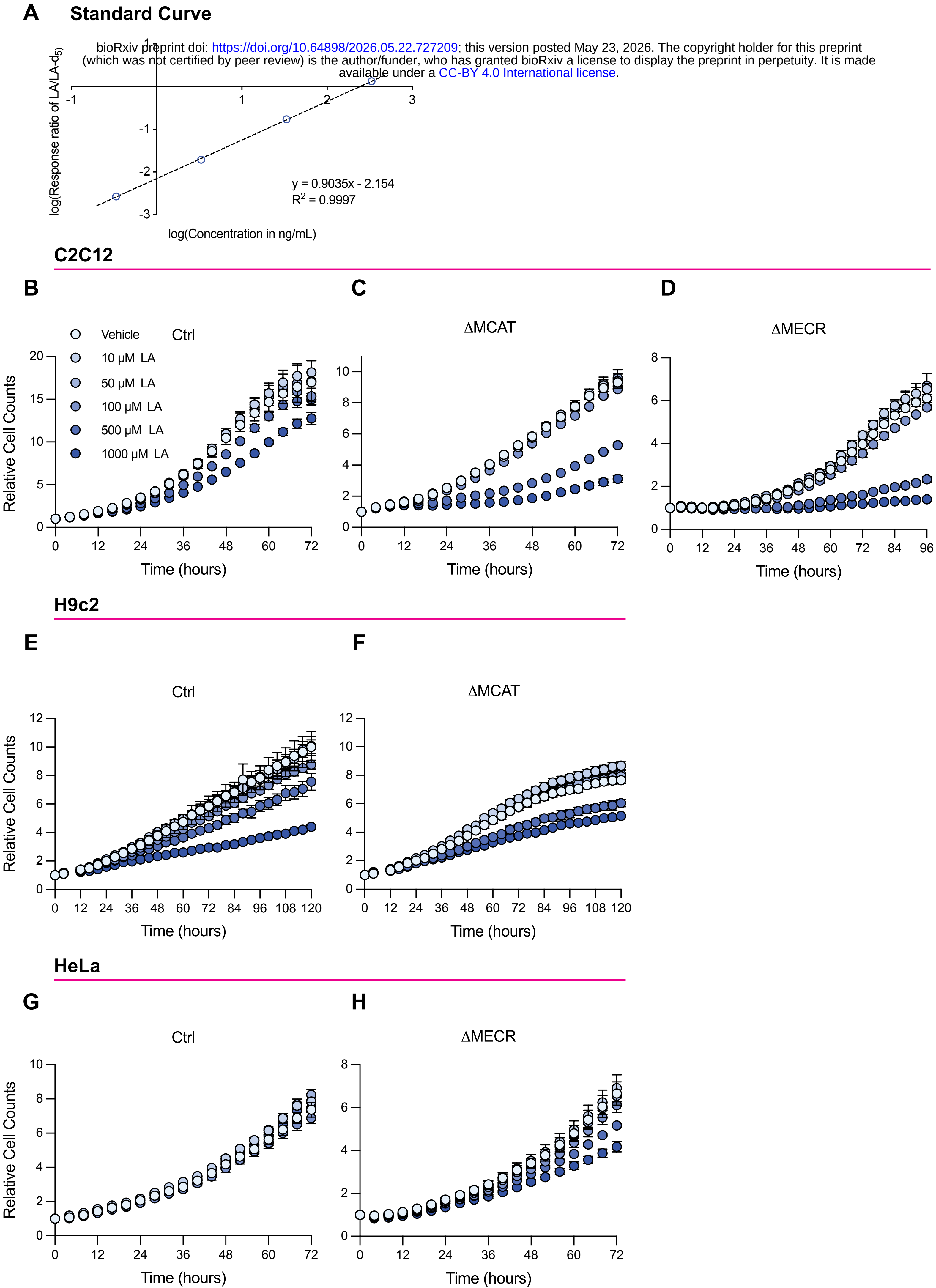

Supplement: 1 [file NIHPP2026.05.22.727209v1-supplement-1.pdf]
